# Urine BMP is lowered in frontotemporal dementia due to progranulin mutations

**DOI:** 10.1002/alz.086871

**Published:** 2025-01-09

**Authors:** Katie Tucker, Imogen J Swift, Sophie E Goldsmith, Aitana Sogorb Esteve, Jonathan D. Rohrer

**Affiliations:** ^1^ Dementia Research Centre, Queen Square Institute of Neurology, University College London, London UK; ^2^ UK Dementia Research Institute at University College London, London UK

## Abstract

**Background:**

GRN mutations are a common cause of frontotemporal dementia (FTD), with previous studies linking granulin deficiency to reduced bis(monoacylglycerol)phosphate (BMP) levels, which ultimately impairs ganglioside degradation. BMP is involved in the lysosomal functions within cells, as it facilitates the adhesion of hydrolases and activator proteins where the lysosomal membranes meet, therefore a lack of BMP could impact lysosome function and integrity. We hypothesised that urine levels of BMP isoforms will be lower in FTD patients with GRN haploinsufficiency, as a reflection of reduced BMP in neural tissues, when compared to those with FTD caused by C9orf72 expansions and MAPT mutations, or healthy controls. The objective of this study was to investigate changes to urinary phospholipids as potential biomarkers of GRN mutations in FTD.

**Method:**

A panel of 3 different di‐22:6 BMP, and 3 di‐18:1 BMP isoforms, as well as total di‐22:6 and di‐18:1 BMP, were measured in urine collected from the UCL GENFI cohort consisting of 36 controls, 11 symptomatic GRN mutation carriers, 11 symptomatic C9orf72 carriers and 12 symptomatic MAPT mutation carriers. A mass spectrometric method was used to quantitate molecular species of BMPs in urine, with results normalised to creatinine. Data analysis was performed in Stata, using an age and sex‐adjusted bootstrapped regression to compare the different symptomatic groups to the controls, with P<0.05 considered statistically significant.

**Result:**

GRN mutation carriers showed reduced levels of the 2:2 di‐22:6 BMP isoform (mean=11.9 (standard deviation=7.5)) when compared to controls (17.9 (13.5); p=0.015), with a trend for reduced levels of the 2:2 di‐18:1 BMP isoform also (3.5 (2.1)) for GRN mutation carriers compared with (4.6 (4.2)) for controls, consistent with the idea that mutations in GRN lower levels of BMP. Concentrations of the 2:3 and 3:3 isoforms as well as the total amount were not significantly different between control and mutation groups.

**Conclusion:**

Our results indicate that GRN mutations cause a reduction in the levels of certain isoforms of BMP in urine. Future studies will be helpful to see if urinary BMP concentrations increase with treatments that restore progranulin levels.

